# Shame or Anger? The Impact of Negative Performance Feedback Sources (AI Versus Leader) on Employees’ Job Crafting

**DOI:** 10.3390/bs16020248

**Published:** 2026-02-09

**Authors:** Ganli Liao, Xiaofeng Ren, Xinyi Zheng, Yuanya Zhang

**Affiliations:** 1 Business School, Beijing Information Science and Technology University, Beijing 100192, China; 2024020827@bistu.edu.cn (X.R.); 2022011293@bistu.edu.cn (Y.Z.); 2 School of Business Administration, Wuhan Business University, Wuhan 430056, China; 20170129@wbu.edu.cn

**Keywords:** leader negative performance feedback, AI negative performance feedback, job crafting, shame, anger, leader trust, algorithm aversion

## Abstract

With the growing adoption of artificial intelligence (AI) in organizational performance management, AI feedback has increasingly supplemented or replaced leader-delivered evaluations. While prior research has addressed issues of fairness and accuracy in AI assessments, relatively little is known about how employees emotionally and behaviorally respond to negative performance feedback (NPF) from different sources. Building on Affective Events Theory, this study investigates how leader versus AI elicits distinct emotions, shame and anger, and how these emotions subsequently influence employees’ job crafting. Two studies were conducted to test the proposed model. Study 1 employed a scenario-based experiment to compare employees’ emotional reactions. Results indicate that leader NPF evokes greater shame, whereas AI NPF induces stronger anger. Study 2 used survey data from nine enterprises in China to further test the underlying mechanisms. Results show that shame and anger mediate the effects of leader and AI NPF on promotion-oriented and prevention-oriented job crafting, respectively. Moreover, leader trust weakens the relationship between leader NPF and shame, while algorithm aversion strengthens the relationship between AI negative feedback and anger. This study advances understanding of the emotional mechanisms underlying employees’ responses to negative feedback and offers practical insights for designing effective human–AI feedback systems in organizations.

## 1. Introduction

The accelerated digitization of organizational practices has profoundly transformed the ways in which employee performance is monitored, assessed, and managed (Leavitt et al., 2024; Kalischko & Riedl, 2021). Notably, the integration of artificial intelligence (AI) and algorithmic decision-making systems is gaining traction as a means of delivering performance feedback, roles previously associated with leaders, though emerging evidence shows that employee responses to AI feedback can differ systematically from responses to human evaluation (Tong et al., 2021; Qin et al., 2023; Hoffmann & Thommes, 2020). Companies such as Unilever and Cogito, for instance, have introduced AI-driven platforms that evaluate employee behaviors, track customer interactions, and generate tailored suggestions for improvement (Marr, 2018; De La Garza, 2019). Similarly, Enaible has developed an AI program that remotely monitors workflows, assigns productivity scores, and recommends efficiency adjustments (Heaven, 2020). Overall, these examples underscore the growing reliance on AI feedback in the workplace, which is commonly justified by its efficiency, objectivity, and broad applicability.

Yet performance feedback is not always positive, namely, negative performance feedback (NPF). This often conveys critical or corrective information that strongly shapes how employees think, feel, and behave at work (Ilgen & Davis, 2000; Belschak & Den Hartog, 2009). Previous studies proposed that negative performance feedback has long been recognized as a means for guiding employee development and enhancing organizational effectiveness (Choi et al., 2018). However, some scholars argued that negative performance feedback also can provoke defensive reactions and undermine motivation. Therefore, scholars have increasingly explored whether negative performance feedback originating from AI elicits distinct employee responses compared with feedback generated by leaders (Tong et al., 2021; Li et al., 2025; Liao et al., 2024). Within this stream of work, leader negative performance feedback has been widely investigated. By contrast, AI negative performance feedback is generated through data-driven processes, often relying on standardized metrics, performance analytics, or algorithmic scoring systems (Pei et al., 2024; Liao et al., 2024).

Regardless of whether negative performance feedback is generated by leaders or AI, existing studies have primarily focused on its behavioral outcomes, such as performance, fairness perceptions, and retreat behaviors (Tong et al., 2021; Eggers & Suh, 2019), while devoting less attention to the emotional mechanisms. In fact, emotions play an essential role in how employees process performance feedback, as they influence not only immediate reactions but also longer-term attitudes and behaviors in the workplace (Van Kleef et al., 2015; Sufi et al., 2024). Among the emotions elicited by negative performance feedback, shame and anger emerge as particularly salient, given their strong implications for employee’s attitudes and behaviors (Bonner et al., 2017; Xing et al., 2021; Gibson & Callister, 2010; Fitness, 2000). This raises a pressing question: how do employees emotionally respond to negative performance feedback when it generates from an AI compared with a leader?

To address this gap, we draw on Affective Events Theory (AET; Weiss & Cropanzano, 1996) to develop a theoretical framework that explains how negative performance feedback from leader vs. AI differentially elicits employees’ shame and anger. According to AET, workplace events give rise to affective responses that subsequently shape employees’ attitudes and behaviors. In the context of performance management, leader negative performance feedback and AI negative performance feedback can be conceptualized as two types of affective events that differ in their social-interactional and data-driven nature, thereby triggering shame and anger in different ways. Moreover, such emotional differences may lead to distinct behavioral outcomes. Specifically, this study focuses on job crafting, which refers to employees’ proactive changes to the task, relational, and cognitive boundaries of their work (Tims et al., 2012; Zhang & Parker, 2019). Prior research suggests that job crafting comprises two dimensions (Bindl et al., 2019; Boehnlein & Baum, 2022). Promotion-oriented job crafting refers to behaviors such as expanding responsibilities, seeking additional resources, or building constructive relationships, whereas prevention-oriented job crafting involves reducing work demands, avoiding challenging tasks, or minimizing exposure to negative interactions (Chen et al., 2024). Given that shame tends to foster an inward focus on self-improvement (Xing et al., 2021), whereas anger reflects an outward focus on blame and resistance (Gibson & Callister, 2010), we propose that negative performance feedback from different sources may give rise to distinct forms of job crafting. Moreover, these emotional mechanisms may vary depending on boundary conditions. From a trust perspective, this study further examines whether leader trust (Burke et al., 2007; Campagna et al., 2020) and algorithm aversion (Jussupow et al., 2020; Mahmud et al., 2022; Burton et al., 2020) moderate the relationship between negative performance feedback and employees’ emotional responses.

This study makes several contributions. First, it advances the feedback literature by demonstrating that the source of negative performance feedback (leader vs. AI) triggers distinct emotional responses—shame and anger—that in turn drive different forms of job crafting. In doing so, it moves beyond the dominant focus on feedback content and fairness to highlight the importance of feedback source as an affective trigger. Second, by integrating Affective Events Theory with the job crafting perspective, this study enriches our understanding of how discrete emotions channel employee reactions into promotion- versus prevention-oriented behaviors, thereby offering a more nuanced account of the feedback–behavior link. Third, this research identifies boundary conditions, namely leader trust and algorithm aversion, that shape the intensity and direction of emotional reactions, extending knowledge on when and why employees accept or resist feedback in digitized contexts. Finally, the findings provide timely insights for organizations navigating the growing use of algorithmic management, underscoring the need to balance efficiency with emotional and motivational employee dynamics in performance evaluation practices. The rest of this study is organized as follows. Section 2 reviews the literature and develops the theoretical framework and hypotheses. Section 3 outlines the overall research methodology. Section 4 and Section 5 present the design and results of Study 1 and Study 2, respectively. Section 6 discusses the theoretical and practical implications of the findings. Finally, Section 7 concludes the paper and highlights limitations and directions for future research.

## 2. Theory and Hypotheses

### 2.1. Impact of Negative Performance Feedback on Employees’ Shame and Anger

Affective Events Theory (AET) (Weiss & Cropanzano, 1996) posits that workplace events trigger affective reactions, which in turn influence employees’ attitudes and behaviors. Negative performance feedback, as a critical workplace event, refers to information indicating that an employee’s performance, behavior, or outcomes fail to meet expected standards (Steelman & Rutkowski, 2004). Although intended to facilitate performance improvement, negative performance feedback frequently provokes strong emotional responses because it highlights a discrepancy between actual and expected performance (Schmeichel & Demaree, 2010; Belschak & Den Hartog, 2009). With the accelerating digitalization of enterprises and the growing integration of AI, negative performance feedback can be delivered either by leaders, who provide evaluations based on interpersonal interactions and subjective judgments (Simon et al., 2022; Ilgen & Davis, 2000; Xing et al., 2023), or by algorithmic systems, which rely on data-driven assessments (Pei et al., 2024; Hoffmann & Thommes, 2020). Due to the distinct characteristics of these feedback sources, they are likely to evoke different emotional responses in employees (Thuillard et al., 2022; Liao et al., 2024; Qin et al., 2023).

Among the emotions elicited by negative performance feedback, shame has received substantial attention (Murphy & Kiffin-Petersen, 2017). Shame is a self-conscious emotion characterized by feelings of personal inadequacy, self-blame, and withdrawal tendencies (Tangney & Dearing, 2003; Tracy & Robins, 2006; Daniels & Robinson, 2019). Empirical studies have demonstrated that negative performance feedback is a significant antecedent of shame in the workplace, which in turn influences job performance and well-being outcomes (Xing et al., 2021; Zada et al., 2022). This study further compares how leader versus AI negative performance feedback differentially influence employees’ experience of shame. We argue that leader negative performance feedback tends to carry stronger social-evaluative implications than AI feedback, making employees more vulnerable to experiencing shame. First, leader negative performance feedback carries strong social-evaluative implications, as they represent the judgment of a salient authority figures whose opinions directly affect employees’ reputation and relational standing within the organization. Such interpersonal scrutiny heightens employees’ awareness of their shortcomings and intensifies negative self-appraisals. Second, leader negative performance feedback is often perceived as intentional and emotionally laden, incorporating tone, wording, and non-verbal cues that convey disapproval. This perceived intentionality strengthens the sense of personal failure beyond task performance. By contrast, AI negative performance feedback is typically framed as impersonal and data-driven, detached from interpersonal motives or affective expressions. Because employees attribute AI negative performance feedback to neutral computational processes rather than to a socially significant evaluator, they are less likely to perceive it as threatening to their self-worth. Thus, we propose that:

**Hypothesis** **1 (H1).** *Compared to AI negative performance feedback, leader negative performance feedback is more strongly associated with employees’ shame.*

Another key emotional reaction to negative performance feedback is anger, an outward-directed emotion that arises when individuals perceive external agents as responsible for unfavorable outcomes or as violating fairness norms (Domagalski & Steelman, 2005; Moreo et al., 2020; Fitness, 2000). Although AI evaluations apply rules consistently and appear impartial, employees often experience them as unfair because the process is non-transparent, insensitive to situational factors, and leaves no room for them to express their views (Shin & Park, 2019; Lee, 2018). We propose that AI negative performance feedback may induce stronger anger than leader negative performance feedback for the following reasons. First, AI-driven assessments typically rely heavily on standardized metrics and quantitative indicators, which, while enhancing formal consistency, also risk being experienced as rigid to situational constraints. This rigidity may foster perceptions of injustice, as employees feel that the complexity of their performance is inadequately recognized. Second, algorithmic processes limit transparency, depriving employees of the ability to understand, challenge, or negotiate the basis of the evaluation. Third, AI negative performance feedback reduces opportunities for interaction, empathy, and justification, as well as interpersonal dynamics that can mitigate frustration and maintain relational fairness in leader negative performance feedback (Lee, 2018; McGuire & De Cremer, 2023). Unlike leaders, who may soften critical feedback through tone, contextual explanations, or recognition of effort, AI lacks the capacity for emotional adjustment. Consequently, employees may interpret AI negative performance feedback as a rigid judgment imposed by an impersonal system, thereby intensifying frustration, resentment, and ultimately anger. Thus, we propose that:

**Hypothesis** **2 (H2).** *Compared to leader negative performance feedback, AI negative performance feedback is more strongly associated with employees’ anger.*

### 2.2. Mediating Effects of Shame and Anger

AET emphasizes that workplace events influence employees’ behaviors primarily through affective reactions (Weiss & Cropanzano, 1996). Negative performance feedback, as an affective event, triggers specific emotional responses that drive employees’ subsequent behavioral strategies. In the context of job design, job crafting is defined as employees’ proactive adjustments to their tasks, relationships, and work cognitions to better align with personal needs and organizational goals (Wrzesniewski & Dutton, 2001; Choi et al., 2018; Belschak & Den Hartog, 2009). It is particularly sensitive to employees’ emotional responses (Z. Wang & Jiang, 2023). Prior research suggests that discrete emotions, in contrast to generalized affect, play different roles in motivating employees to engage in constructive or defensive forms of job crafting (Bakker et al., 2020; Bindl et al., 2019). Building on this perspective, we argue that shame and anger, two distinct emotions elicited by different negative performance feedback sources, serve as critical mediators linking negative performance feedback to divergent forms of job crafting.

Shame, as a self-conscious emotion, often arises when individuals perceive themselves as failing to meet socially or professionally significant standards (Daniels & Robinson, 2019). Although shame is inherently aversive, it can also promote constructive behaviors aimed at restoring one’s social image and self-worth (De Hooge et al., 2011). In organizational contexts, employees experiencing shame often engage in self-improvement efforts to counteract the negative implications of their perceived shortcomings (Stuewig & Tangney, 2007). Promotion-oriented job crafting provides a strategy for such restorative action, as it enables employees to actively seek resources, expand capabilities, and reframe work in ways that enhance competence and performance (Wrzesniewski & Dutton, 2001; Q. Wang et al., 2025; Barclay et al., 2022). Thus, when employees feel shame following leader negative performance feedback, they may be more inclined to engage in promotion-oriented job crafting as a means to regain a positive professional identity. Compared to AI, leader negative performance feedback is particularly salient in evoking shame because it originates from a social source, carrying implicit judgments about one’s competence and value within the team (Mawritz et al., 2014; Simon et al., 2022). Employees tend to internalize leader feedback, heightening self-awareness and strengthening the motivation to restore lost standing through proactive work behaviors (Belschak & Den Hartog, 2009; Wu & Parker, 2017; Fuller et al., 2015). Accordingly, we propose that:

**Hypothesis** **3 (H3).** *Shame mediates the relationship between leader negative performance feedback and the employees’ promotion-oriented job crafting.*

Anger is an outward-directed, action-oriented emotion that arises when individuals attribute negative outcomes to external, unfair, or uncontrollable sources (Lazarus, 1991; Berkowitz & Harmon-Jones, 2004). AI negative performance feedback is particularly likely to elicit anger since it is perceived as impersonal, rigid, and decontextualized, thereby limiting employees’ sense of control and fairness (Shin & Park, 2019). When employees experience anger, they are motivated to protect themselves from further perceived threats or injustices. This protective motivation is reflected in prevention-oriented job crafting, in which employees proactively adjust their work to reduce exposure to risk, avoid challenging or high-demand tasks, and limit engagement with evaluative processes. In this way, anger generates a heightened sensitivity to potential threats in the work environment, prompting employees to adopt defensive strategies that preserve their resources, minimize further losses, and maintain a sense of control over their workload. AI negative performance feedback, despite being formally objective, tends to heighten perceptions of unfairness and helplessness because it lacks transparency, contextual sensitivity, and opportunities for communication. These features make employees more prone to anger, which subsequently increases prevention-oriented job crafting. Thus, we propose that:

**Hypothesis** **4 (H4).** *Anger mediates the relationship between the AI negative performance feedback and employees’ prevention-oriented job crafting.*

### 2.3. Moderating Effect of Leaders Trust and Algorithm Aversion

Although negative performance feedback elicits discrete emotional responses that influence job crafting, the intensity and direction of these effects depend on employees’ perceptions and attitudes toward the feedback source. According to AET, affective reactions are shaped not only by the characteristics of the triggering event but also by individual-level appraisals, such as perceived fairness, legitimacy, and organizational context (Weiss & Cropanzano, 1996; Matta et al., 2014; Barsky et al., 2011). Therefore, this study further examines whether leader trust and algorithm aversion moderate the relationship between negative performance feedback and employees’ emotional reactions, and subsequently exert indirect effects on job crafting through these emotions.

Leader trust reflects employees’ belief in the leader’s competence, benevolence, and integrity (Joseph & Winston, 2005; Campagna et al., 2020). Prior research shows that leader trust fundamentally shapes how employees interpret and respond to managerial actions (Burke et al., 2007; Islam et al., 2021). High leader trust facilitates positive appraisals of feedback, such that negative evaluations are interpreted as constructive and developmental rather than punitive (Colquitt et al., 2007). When employees trust their leader, they are more likely to attribute performance shortcomings to controllable factors and perceive negative performance feedback as a guidance opportunity rather than a personal threat. In such contexts, the experience of shame is more likely to be channeled into constructive outcomes, motivating employees to engage in promotion-oriented job crafting as a way to restore competence and meet expectations. Conversely, when leader trust is low, employees may question the fairness or intentions behind negative performance feedback, which weakens the link between negative performance feedback and shame. These attributions further undermine the adaptive potential of shame, reducing its likelihood of translating into proactive work adjustments and instead fostering defensive reactions or withdrawal. Thus, we propose that:

**Hypothesis** **5 (H5).** *Leader trust negatively moderates the relationship between leader negative performance feedback and employees’ shame, such that the positive relationship is weaker when leader trust is higher.*

Algorithm aversion refers to individuals’ tendency to distrust or resist algorithmic decision-making, especially in contexts involving subjective judgments or fairness concerns (Dietvorst et al., 2015; Burton et al., 2020; Mahmud et al., 2022). When employees are high in algorithm aversion, they are more likely to interpret AI negative feedback as unfair, rigid, and lacking legitimacy (Liao et al., 2024). This amplifies negative emotional reactions—employee anger—because employees attribute unfavorable evaluations to an impersonal and insensitive system that deprives employees of opportunities for interaction or recourse. Moreover, high algorithm aversion can be understood through the lens of perceived controllability and fairness, when employees are highly averse to AI, they appraise negative performance feedback as externally imposed and unjust, which heightens anger and motivates prevention-oriented job crafting. In contrast, employees low in algorithm aversion may perceive AI feedback as relatively objective and consistent, thereby reducing the intensity of anger elicited by such evaluations. Thus, we propose that:

**Hypothesis** **6 (H6).** *Algorithm aversion positively moderates the relationship between AI negative performance feedback and employees’ anger, such that the positive relationship is stronger when algorithm aversion is higher.*

## 3. Research Methodology

**Figure 1:**
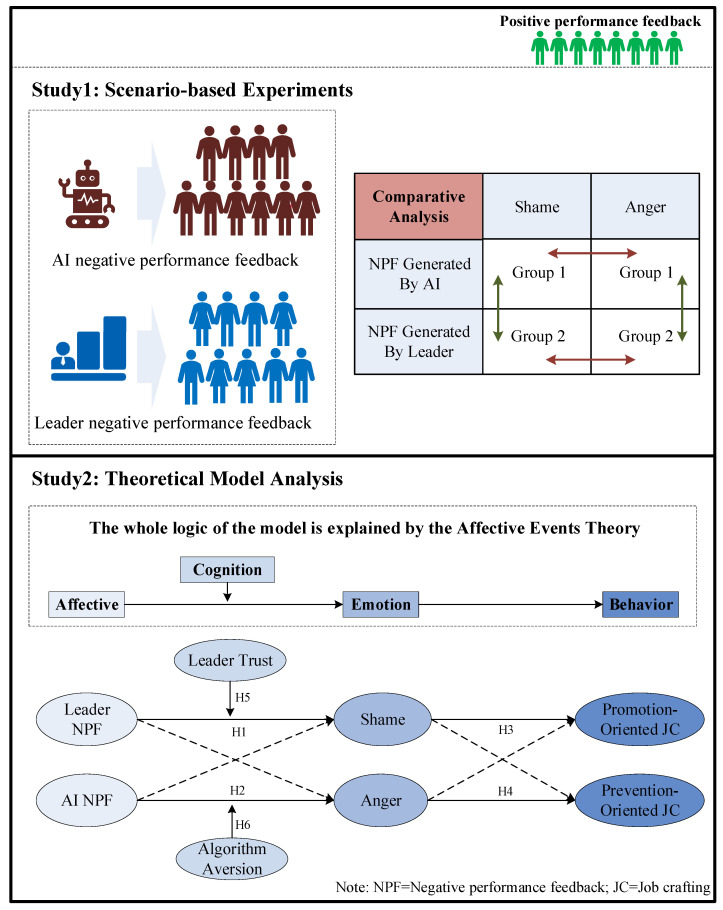
Theoretical model (source: made by authors).

This research employed a multi-study design to investigate how leader vs. AI negative performance feedback influences employees’ emotional responses and job crafting. Study 1 employed a scenario-based experimental design, manipulating negative performance feedback sources to examine participants’ emotional reactions—specifically shame and anger. This design allowed for controlled observation of how different feedback sources are associated with distinct emotional responses. Study 2 adopted a survey-based field approach to explore these mechanisms in actual organizational settings. Employees reported on their experiences with leader vs. AI negative performance feedback, emotional responses, and job crafting. This study complements Study 1 by assessing whether results observed in the experimental scenarios are reflected in real workplace contexts. In sum, the two studies provide a comprehensive overview of the research framework, combining controlled scenario manipulations with field data to capture the dynamics of feedback, emotions, and job crafting. The theoretical model is shown in Figure 1.

## 4. Study 1

### 4.1. Scenario-Based Experiment

This study employed a scenario-based experimental approach (Kim & Jang, 2014) and designed a series of negative feedback scenarios to examine whether different sources of feedback would lead to distinct emotional reactions. The experimental materials consisted of scenario descriptions involving negative performance feedback. Both scenarios were presented from a third-person perspective to avoid directly asking participants about their emotional reactions, thereby reducing social desirability bias and enhancing the external validity of the scenarios.

The experiment adopted a scenario-based design (negative feedback source: leader vs. AI). Each scenario described employees’ failure to meet work expectations, specifically under-performance in task completion. The feedback source was manipulated through two groups: “leader negative performance feedback” and “AI negative performance feedback.” The leader group emphasized personalized and interpersonal evaluation, highlighting leaders’ assessment of employees’ performance. In contrast, the AI group emphasized systematized, data-driven evaluation. The scenarios were constructed as follows:

The reading materials for both the leader and the AI group were identical across conditions, with the only variation being the source of negative performance feedback. Li Ming for the leader group and the “AX-300” AI evaluation system for the AI group. Refer to Appendix A for more details of reading materials.

After reading the scenario materials, participants were asked to complete a questionnaire. This questionnaire measured two variables, shame and anger, using well-validated scales that have been widely applied in top journals. Shame was assessed with a 4-item scale developed by Watson et al. (1996), which we adapted into a situational format to fit the research design. Specifically, participants were asked to evaluate the extent to which the focal character, “Chen Yu,” would experience each affective state. A sample item is, “Chen Yu would feel embarrassed.” The Cronbach’s α for this scale was 0.75. Anger was measured with a 3-item scale from the same source, with a representative item such as, “Chen Yu would feel upset.” The Cronbach’s α for this scale was 0.83, indicating good internal consistency. All items were rated on a 5-point Likert scale, ranging from “1 = strongly disagree” to “5 = strongly agree”.

### 4.2. Manipulations and Samples

The required sample size for this experiment was calculated using G*Power 3.1. For an independent samples t-test, assuming a medium effect size (d = 0.5) and a significance level (α = 0.05), the results indicated that a minimum of 172 participants would be needed to achieve 90% statistical power. Data were collected between November 2024 and January 2025. During this period, participants were recruited through Credamo (https://www.credamo.com/, accessed on 8 November 2024), a professional online data collection platform in China. Eligibility criteria required participants to be at least 20 years old, employed full-time, and to maintain a questionnaire completion rate of over 90%. All participants were randomly assigned to one of two groups and asked to read the corresponding scenario materials. Then, they completed the questionnaire, which measured the extent to which they experienced shame and anger in the given scenario.

In total, 350 questionnaires were distributed. After excluding 47 questionnaires that failed the attention check, 303 valid responses were obtained, yielding a response rate of 86.57%. An additional 38 questionnaires were removed due to missing data, excessive repetitive responses, careless answering patterns, or regular patterned responses. The final dataset consisted of 265 participants (overall valid response rate = 75.71%). Among them, 139 were male (52.45%) and 126 were female (47.54%). In terms of age distribution, 34 participants (12.83%) were between 20 and 25 years old, 101 (38.11%) were between 26 and 35, 78 (29.43%) were between 36 and 45, 32 (12.08%) were between 46 and 55, and 20 (7.55%) were above 55 years. Regarding educational background, 57 participants (21.51%) held a junior college degree or below, 156 (58.87%) had a bachelor’s degree, and 52 (19.62%) possessed a master’s degree or higher. With respect to organizational tenure, 62 participants (23.40%) reported less than 3 years of work experience, 106 (40.00%) had 3–10 years, 61 (23.02%) had 11–20 years, 21 (7.92%) had 21–25 years, and 15 (5.66%) reported more than 26 years of experience. In terms of marital status, 120 participants (45.28%) were unmarried, 111 (41.89%) were married, and 34 (12.83%) reported other statuses. Participants were distributed across a wide range of industries, ensuring variability in occupational contexts. Of the total sample, 141 participants were assigned to the leader negative performance feedback group and 124 participants to the AI negative performance feedback group. All participants voluntarily took part in the study, provided informed consent, and received appropriate compensation upon completion.

### 4.3. Experimental Results

**Figure 2:**
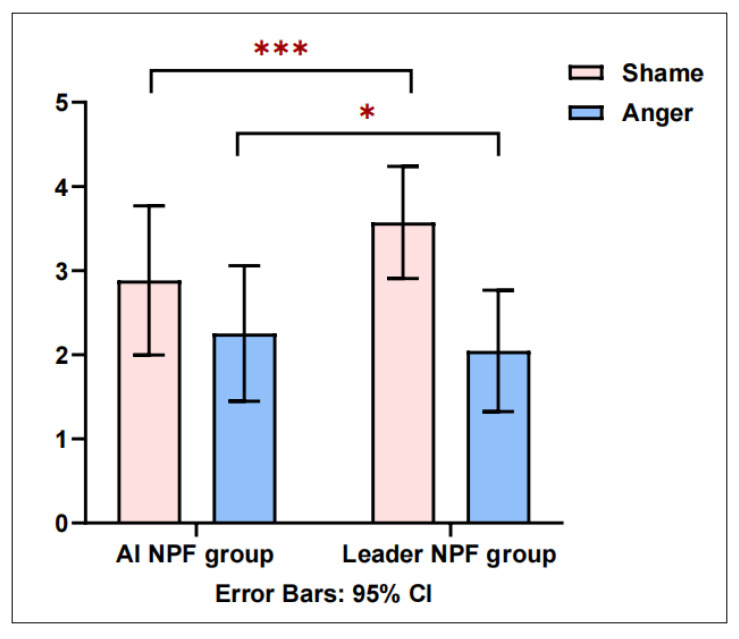
Results of *t*-tests comparing employees’ shame and anger between the AI NPF group and the leader NPF group (* *p* < 0.05, *** *p* < 0.001; source: made by authors).

Independent sample *t*-tests were conducted using GraphPad Prism 10.1 to examine the differences between the AI negative performance feedback group and the leader negative performance feedback group. As shown in Figure 2, employees in the AI negative performance feedback group reported significantly lower levels of shame (Mean = 2.883, SD = 0.885) compared with those in the leader negative performance feedback group (Mean = 3.573, SD = 0.667), t = −7.086, *p* < 0.001, Cohen’s d = 0.777. However, employees in the AI negative performance feedback group reported significantly higher levels of anger (Mean = 2.253, SD = 0.806) than those in the leader negative performance feedback group (Mean = 2.045, SD = 0.722), t = 2.214, *p* < 0.05, Cohen’s d = 0.762. To further assess the joint effects of feedback source on employees’ emotional responses, a multivariate analysis of variance was conducted with shame and anger as dependent variables. Results indicated a significant main effect of feedback source (Wilks’ λ = 0.813, F = 30.198, *p* < 0.001, η^2^ = 0.187). These findings suggest that the source of negative performance feedback (leader vs. AI) exerts a significant influence on employees’ shame and anger. Therefore, H1 and H2 were supported.

## 5. Study 2

### 5.1. Measures

Study 2 employed a survey-based research design to further investigate the mechanisms through which different sources of negative performance feedback influence employees’ emotions and behaviors. All constructs were measured using well-established scales published in top-tier international journals. Responses were recorded on a 5-point Likert scale, ranging from “1 = strongly disagree” to “5 = strongly agree”. To ensure measurement validity and accuracy, the original English items were translated into Chinese following a double-blind translation and back-translation procedure. Subsequently, 4 professors in management and 3 doctoral candidates specializing in organizational behavior were invited to review the translated items. Their feedback was incorporated to refine wording and guarantee contextual appropriateness for Chinese respondents.

Negative performance feedback. Leader negative performance feedback was measured using the 4-item scale developed by Steelman & Rutkowski (2004). A representative item is “When I do not complete a task on time, my leader lets me know.” The Cronbach’s α of this scale was 0.903, suggesting high internal consistency. To measure AI negative performance feedback, the same items were adapted by replacing the feedback source with an AI. For example: “When I do not complete a task on time, the organization’s performance feedback system/AI reminds me.” The Cronbach’s α of this scale was 0.893, demonstrating good reliability and validity.

Shame and anger. The measures for shame and anger were identical to those used in Study 1, adapted from Watson et al. (1996). In the present study, shame (4 items) yielded a Cronbach’s α of 0.810, while anger (3 items) yielded a Cronbach’s α of 0.824.

Job crafting. This was measured using the validated scale developed by Tims et al. (2012), which has been widely applied in organizational research. Promotion-focused job crafting includes 15 items in total. Representative items include: “I make an effort to improve my job-related abilities,” “I ask my colleagues for feedback,” and “When an interesting project arises, I proactively request to be involved.” Prevention-focused job crafting was measured with 6 items, such as: “I try to reduce the emotional demands of my work” and “I organize my work so as to avoid continuous strain.” The Cronbach’s α were 0.928 and 0.907, respectively.

Algorithm aversion. Based on previous research (Liao et al., 2024), algorithm aversion was assessed across three dimensions: permissibility, liking, and utilization intention. Permissibility was measured using the scale developed by Bigman and Gray (2018); liking for algorithms was measured using Jago’s (2019) scale; and utilization intention was measured with items from Cadario et al. (2021). The overall scale comprised 6 items, such as: “Is the appraisal decision made by the algorithm system appropriate?” The Cronbach’s α of the scale was 0.716.

Leader trust. This was measured using the 5-item scale by Podsakoff et al. (1990). A representative item is: “I completely trust my leader to be honest and truthful.” The Cronbach’s α of the scale was 0.764.

Control variables. Prior research suggests that employees’ demographic characteristics, such as education, gender, and age, may influence their emotions and work behaviors. To alleviate potential bias, this study included gender, age, education, marriage, and working years as controlled variables.

### 5.2. Samples

Data for Study 2 were collected between February 2025 and June 2025 through WeChat and email surveys distributed across 9 enterprises located in Beijing, Guangdong, Jiangxi, and Hebei provinces. This multi-site data collection strategy ensured diversity in both industry background and regional distribution. Prior to distributing the questionnaires, we contacted the human resources directors of each company to explain the academic purpose of the study and to obtain organizational consent. The HR directors assisted in identifying full-time employees and in disseminating the survey link internally. All participants were informed of the voluntary nature of participation and were assured of the anonymity and confidentiality of their responses.

A total of 732 questionnaires were distributed. After excluding 28 questionnaires that failed attention checks and 45 questionnaires with excessive missing data, 659 valid responses were retained, yielding an effective response rate of 90.0%. The final sample consisted of 349 males (52.95%) and 310 females (47.05%). Regarding age, 109 participants (16.54%) were between 20 and 25 years old, 267 (40.52%) were between 26 and 35, 168 (25.49%) were between 36 and 45, 85 (12.90%) were between 46 and 55, and 30 (4.55%) were above 55 years old. With respect to educational background, 191 participants (28.98%) held an associate degree or below, 327 (49.62%) held a bachelor’s degree, and 141 (21.40%) possessed a master’s degree or higher. In terms of organizational tenure, 106 participants (16.09%) had less than 3 years of work experience, 317 (48.11%) had 3–10 years, 131 (19.88%) had 11–20 years, 68 (10.32%) had 21–25 years, and 37 (5.61%) had more than 26 years. Concerning marital status, 314 participants (47.65%) were unmarried, 275 (41.73%) were married, and 70 (10.62%) were categorized as other. All respondents voluntarily participated and received modest compensation upon survey completion.

### 5.3. Descriptive Analysis

Descriptive and correlation analysis were conducted using SPSS 27.0. The means, standard deviations (SD), and correlation coefficients of all variables are presented in Table 1. Results indicated that leader negative performance feedback and shame were both positively associated with promotion-oriented job crafting (r = 0.669, r = 0.862, *p* < 0.01). Leader negative performance feedback was positively related to shame (r = 0.549, *p* < 0.01). In addition, AI negative performance feedback and anger were both positively associated with prevention-oriented job crafting (r = 0.325, r = 0.880, *p* < 0.01). Algorithm aversion was negatively associated with prevention-oriented job crafting (r = −0.254, *p* < 0.01), while AI negative performance feedback was positively related to anger (r = 0.326, *p* < 0.01). These results provide preliminary evidence for the proposed model.

### 5.4. Confirmatory Factor Analyses

To assess the discriminant validity of the theoretical model, confirmatory factor analyses (CFA) were conducted using Mplus 8.3. As shown in Table 2, a four-factor model consisting of leader negative performance feedback, shame, leader trust, and promotion-oriented job crafting was developed. The results indicated that the four-factors model 1 (*χ*^2^/*df* = 2.963, CFI = 0.914, TLI = 0.894, RMSEA = 0.060, SRMR = 0.131) demonstrates the best fit indices compared to other alternative models. For instance, the one-factor model 1 (all items combined into one single factor) demonstrated a poorer fit (*χ*^2^/*df* = 9.192, CFI = 0.815, TLI = 0.774, RMSEA = 0.111, SRMR = 0.128), as did the two-factor model 1 (*χ*^2^/*df* = 7.342, CFI = 0.857, TLI = 0.825, RMSEA = 0.098, SRMR = 0.128).

Similarly, the four-factors model 2 including AI negative performance feedback, anger, algorithm aversion, and prevention-oriented job crafting was developed. As reported in Table 3, this four-factor model 2 showed good fit indices (*χ*^2^/*df* = 2.851, CFI = 0.942, TLI = 0.933, RMSEA = 0.059, SRMR = 0.046), which were significantly better than those of the alternative models (e.g., three-factors model 3: *χ*^2^/*df* = 12.687, CFI = 0.787, TLI = 0.754, RMSEA = 0.133, SRMR = 0.128). In sum, these results provide strong evidence of discriminant validity among the study variables.

### 5.5. Tests of Hypotheses

This study employed hierarchical regression analyses using PROCESS in SPSS 27.0 to test Hypotheses 3 through 6. The results are presented in Table 4 and Table 5. After controlling the demographic variables, the results in Models 2 and 5 (Table 4) demonstrated that leader negative performance feedback was significantly positively associated with shame ($\beta_{1}$ = 0.557, *p* < 0.001) and promotion-oriented job crafting ($\beta_{2}$ = 0.672, *p* < 0.001), respectively. Model 6 further revealed that, after controlling for shame, the effect of leader negative performance feedback on promotion-oriented job crafting was reduced ($\beta_{3}$ = 0.282, *p* < 0.001). Thus, H3 was supported. In Table 5, after controlling for demographic variables, Models 8 and 11 showed that AI negative performance feedback was significantly positively related to anger ($\beta_{4}$ = 0.282, *p* < 0.001) and prevention-oriented job crafting ($\beta_{5}$ = 0.281, *p* < 0.001). The results in Model 12 demonstrated that, after controlling for anger, the relationship between AI negative performance feedback and prevention-oriented job crafting was insignificant ($\beta_{6}$ = 0.038, *p* > 0.05), indicating that anger fully mediated the relationship. Thus, H4 was supported.

In order to estimate the moderating roles of leader trust and algorithm aversion, interaction terms were constructed in Model 3 and Model 6. The results in Model 3 showed that the interaction between leader negative performance feedback and leader trust was significantly negatively associated with shame ($\beta_{7}$ = −0.402, *p* < 0.001). Model 9 demonstrated that the interaction between AI negative performance feedback and algorithm aversion was significantly positively associated with anger ($\beta_{8}$ = 0.239, *p* < 0.001). Thus, H5 and H6 were supported.

**Figure 3:**
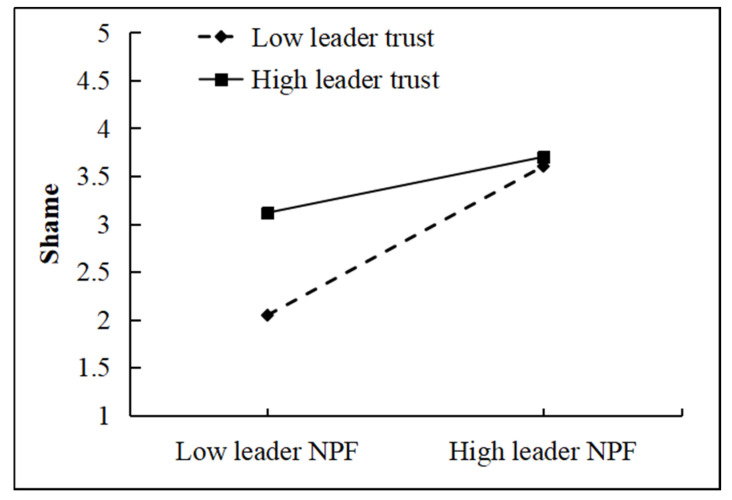
The moderating effects of leader trust between leader NPF and shame (source: made by authors).

This study employed simple slope analyses (Aiken et al., 1991) to examine the moderating effects of leader trust and algorithm aversion at different levels. As illustrated in Figure 3, compared with low leader trust (Mean − 1 SD), high leader trust (Mean + 1 SD) significantly weakened the positive effect of leader negative feedback on shame. Thus, H5 was further supported. In addition, Hayes and Scharkow (2013)’s bootstrap method (=5000 times) was applied to test the conditional indirect effect of shame in mediating the relationship between leader negative performance feedback and promotion-focused job crafting across varying levels of leader trust. As shown in Table 6, the mediating effects of shame were 0.453, 0.311, and 0.170 under low (Mean − 1 SD), medium (Mean), and high (Mean + 1 SD) leader trust, respectively. The corresponding 95% confidence intervals were [0.397, 0.510], [0.264, 0.355], and [0.112, 0.223]. These results indicate that leader negative performance feedback increases employees’ shame, but this positive relationship is significantly weakened at higher levels of leader trust. Moreover, the positive indirect effect of leader negative performance feedback on promotion-focused job crafting through employees’ shame also decreases when leader trust is high.

**Figure 4:**
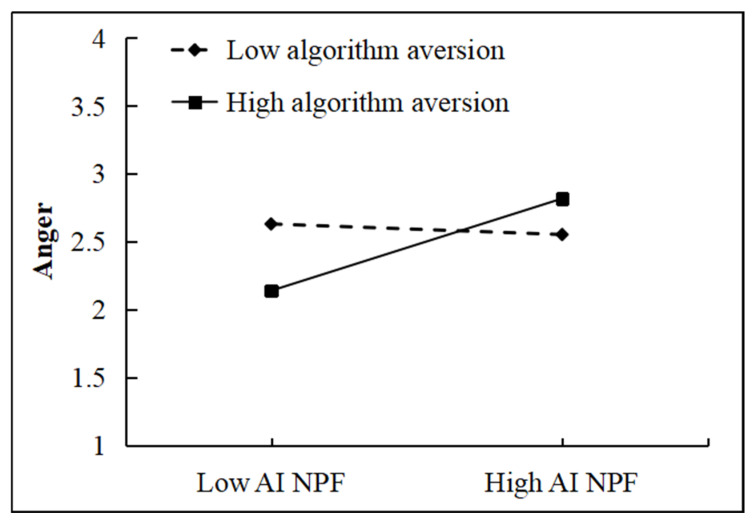
The moderating effects of algorithm aversion between AI NPF and anger (source: made by authors).

As shown in Figure 4, compared to low algorithm aversion (Mean − 1 SD), high algorithm aversion (Mean + 1 SD) significantly strengthened the positive effect of AI negative performance feedback on anger. Thus, H6 was supported. Furthermore, the conditional indirect effect of anger in mediating the relationship between AI negative performance feedback and prevention-oriented job crafting was examined under different levels of algorithm aversion (Table 7). The results demonstrated that the mediating effects of anger were −0.033, 0.129, and 0.290 under low (Mean − 1 SD), medium (Mean), and high (Mean + 1 SD) algorithm aversion, respectively, with the 95% confidence intervals being [−0.137, 0.067], [0.044, 0.209], and [0.199, 0.378]. These findings suggest that AI negative performance feedback elicits employees’ anger, and this effect intensifies as the degree of algorithm aversion increases. In addition, the indirect effect of AI negative performance feedback on prevention-oriented job crafting through employees’ anger is also increased under higher levels of algorithm aversion.

## 6. Discussion

### 6.1. Theoretical Implications

This research makes several theoretical contributions to the literature on negative performance feedback, emotions, and job crafting in the digital era. First, this study advances the literature on feedback and algorithmic management by generating new insights into employees’ perceptual differences toward different feedback sources, namely, leader vs. AI negative performance feedback. While prior research on negative feedback has primarily emphasized informational characteristics such as accuracy, fairness, and credibility (Tong et al., 2021; Newman et al., 2020), relatively little is known about how employees evaluate the perceived appropriateness of the negative feedback and its emotional consequences. Our findings extend this line of work to reveal that employees attribute leader negative performance feedback to interpersonal evaluation, thereby experiencing heightened shame, whereas AI negative performance feedback is perceived as less relational but more detrimental to employees’ confidence in their ability to meet performance expectations, thereby increasing anger. This study aligns with existing scholarship indicating that leader negative performance feedback tends to elicit stronger feelings of shame in employees (Li et al., 2025; Xing et al., 2021). This distinction contributes to a deeper theoretical understanding of how feedback source influences discrete emotional responses. Moreover, it extends the literature on algorithmic management by positioning AI feedback not merely as a technical tool (Olan et al., 2022; Agrawal et al., 2019), but as a social signal that triggers psychological processes.

Second, building on AET, this study enriches the job crafting literature by identifying discrete emotions as psychological mechanisms that translate the impact of negative performance feedback (leader vs. AI) into divergent behavioral strategies. Previous studies have mainly focused on cognitive factors like appraisal, self-regulation (Zhang & Parker, 2022; Bakker & Oerlemans, 2019; Demerouti et al., 2024) or motivational orientations (Niessen et al., 2016; Rofcanin et al., 2019) as the drivers of job crafting (Zhang & Parker, 2019; Lazazzara et al., 2020; Lichtenthaler & Fischbach, 2019), while the role of emotions has been less systematically examined. Our results demonstrate that shame, typically triggered by leader negative performance feedback, facilitates promotion-oriented job crafting by motivating employees to restore competence and maintain a positive professional identity. In contrast, anger, often elicited by AI negative performance feedback, promotes prevention-oriented job crafting by encouraging employees to adopt defensive strategies to reduce risk and protect resources. Building on prior research that has predominantly examined single-source feedback (Cheng et al., 2023; He et al., 2024; Liu et al., 2024), this study uncovers the differentiated mechanisms through which distinct feedback sources influence job crafting. By highlighting the mediating role of shame and anger, it provides a nuanced understanding of why the different sources of negative performance feedback can lead to either promotion-oriented or prevention-oriented job crafting, thereby enriching existing perspectives that have primarily focused on cognitive or motivational processes.

Finally, by incorporating leader trust and algorithm aversion as boundary conditions, this study advances the feedback and emotion literature by specifying for whom negative performance feedback exerts stronger or weaker effects. Prior research has predominantly focused on the direct influence of negative performance feedback (Qin et al., 2023; Pei et al., 2024), with relatively limited attention given to how AI-related or leader-related factors moderated the link between feedback sources and employee behavior (Li et al., 2025; Liao et al., 2024). Extending this stream of research, the present study highlights that employees’ trust in leaders or in AI would influence their emotional and behavioral responses. The results indicate that leader trust weakens the mediating role of shame between leader feedback and promotion-oriented job crafting, whereas algorithm aversion strengthens the mediating role of anger between AI feedback and prevention-oriented job crafting. This perspective not only enriches research on negative performance feedback and job crafting but also contributes to the emerging literature on algorithmic management by revealing how employees’ trust, whether directed toward leaders or toward algorithmic systems, shapes their reactions to leader versus AI sources of authority.

### 6.2. Practical Implications

This research also provides several practical insights for organizational feedback practices in the era of digitalization. First, the findings suggest that organizations should strategically align the source of negative feedback with their intended outcomes. Leader negative performance feedback, although often evoking shame, can transform this emotion into constructive motivation, thereby encouraging promotion-oriented job crafting aimed at enhancing competence and performance. Accordingly, leaders may consider delivering critical feedback personally when the objective is employee development and growth. In contrast, AI negative performance feedback is more likely to elicit anger, an outward-directed emotion associated with perceived unfairness and lack of control, which may foster defensive, prevention-oriented job crafting. Organizations should therefore be cautious about over-reliance on AI-based feedback systems, particularly in sensitive performance evaluation contexts.

Second, the results highlight the importance of cultivating leader trust as a critical buffer in feedback processes. When employees trust their leaders, the shame evoked by negative performance feedback is weakened, reducing the risk that it becomes harmful while preserving its motivational benefits. In practice, this suggests that organizations should invest in leadership development programs that emphasize not only technical competence but also interpersonal credibility, integrity, and fairness. Specifically, training interventions that enhance leaders’ communication skills, empathy, and relational transparency can strengthen trust with subordinates, thereby ensuring that feedback is interpreted as supportive rather than punitive. Moreover, organizational cultures that promote psychological safety, fairness, and consistent leader behavior may further reinforce employee trust, thereby facilitating the constructive reception of negative feedback.

Third, when implementing AI performance management systems, organizations must proactively address employees’ algorithm aversion. Because AI feedback is often perceived as rigid, opaque, and detached from contextual nuances, employees may experience anger and defensiveness in response to such feedback. To mitigate these risks, organizations should increase transparency by clearly communicating the logic, criteria, and data sources underlying AI assessments. It is equally important to provide opportunities for employees to share opinions and seek explanations. These methods not only enhance perceptions of procedural justice but also empower employees with a sense of agency, thereby reducing negative emotional reactions. Such practices may lessen resistance to AI negative performance feedback and strengthen perceived fairness, which in turn increases employees’ willingness to accept and learn from AI feedback.

## 7. Conclusions

### 7.1. Summary of Findings

This research shows that the source of negative performance feedback (leader vs. AI) is associated with distinct discrete emotional reactions. Specifically, leader negative performance feedback is more strongly associated with employees’ shame, whereas AI negative performance feedback is more strongly associated with employees’ anger. These emotions are related to different forms of job crafting: shame is associated with promotion-oriented job crafting, while anger is associated with prevention-oriented job crafting. Moreover, leader trust moderates the positive relationship between leader negative performance feedback and employees’ shame, whereas algorithm aversion amplifies the positive relationship between AI negative performance feedback and employees’ anger.

### 7.2. Limitations and Future Directions

Despite its contributions, this research is subject to several limitations for future investigation. First, the studies primarily focused on shame and anger as the two core emotional reactions to negative feedback. While these emotions are theoretically and empirically central to the feedback–job crafting linkage, employees may also experience other affective states such as guilty, embarrassment, or anxiety (Hershcovis et al., 2017; Ilies et al., 2013). Future research could adopt a broader affective lens to examine whether different emotions shape employees’ behavioral strategies, thereby offering a more nuanced understanding of the emotional dynamics of feedback.

Second, this research relied on a scenario-based experiment (Study 1) and a survey-based field study (Study 2). Although this multi-method design enhances internal and external validity, both approaches have inherent limitations. Experimental scenarios may not fully capture the richness of real workplace interactions, while self-reported survey data may be affected by common method bias or social desirability concerns (Podsakoff et al., 2003; Holtgraves, 2004). To further strengthen external validity, future studies could conduct field experiments in real organizational settings. Moreover, multi-source data collection (e.g., leader evaluations, peer reports, and behavioral observations) or longitudinal and experience-sampling designs could also be conducted to better capture employees’ emotional and behavioral responses to feedback.

Finally, this study examined leader trust and algorithm aversion as boundary conditions. Although these moderators are highly relevant, they represent only a subset of potential factors influencing employees’ responses to feedback. Future research could explore additional moderators, such as personality traits, organizational climate, or the technological features of AI systems. Investigating these factors would enrich understanding of when and for whom negative feedback produces beneficial vs. detrimental outcomes.

## Figures and Tables

**Table 1:** Results of descriptive analysis.

Variables	1	2	3	4	5	6	7	8
1.AI NPF	0.893							
2.Leader NPF	−0.581 **	0.903						
3.Shame	−0.479 **	0.549 **	0.810					
4.Anger	0.326 **	−0.336 **	−0.211 **	0.824				
5.Leader trust	0.623 **	−0.543 **	−0.232 **	0.395 **	0.764			
6.Algorithm aversion	−0.596 **	0.699 **	0.441 **	−0.249 **	−0.399 **	0.716		
7.Pro-JC	−0.583 **	0.669 **	0.862 **	−0.254 **	−0.293 **	0.530 **	0.928	
8.Pre-JC	0.325 **	−0.370 **	−0.214 **	0.880 **	0.409 **	−0.254 **	−0.261 **	0.907
Mean	3.164	2.731	3.252	2.424	3.336	2.963	3.156	2.450
SD	0.974	1.052	0.939	0.941	0.845	0.508	0.822	0.908

Note: ** *p* < 0.01; NPF = negative performance feedback, Pro-JC = promotion-oriented job crafting, Pre-JC = prevention-oriented job crafting, Cronbach’s α coefficients are reported along the diagonal. Source: made by authors according to the questionnaires.

**Table 2:** Results of CFA for leader negative performance feedback model.

Models	Factors	$\chi^{2}$/*df*	CFI	TLI	RMSEA	SRMR
Four-factors model 1	Leader NPF, Shame, Leader trust, Pro-JC	2.963	0.914	0.894	0.060	0.131
Three-factors model 1	Leader NPF + Shame, Leader trust, Pro-JC	6.290	0.882	0.854	0.090	0.162
Three-factors model 2	Leader NPF + Pro-JC, Shame, Leader trust,	7.088	0.864	0.832	0.096	0.157
Two-factors model 1	Leader NPF + Leader trust, Shame, Pro-JC	7.342	0.857	0.825	0.098	0.128
Two-factors model 2	Leader NPF + Shame, Leader trust + Pro-JC	8.543	0.830	0.792	0.107	0.134
One-factor model 1	Leader NPF + Shame + Leader trust + Pro-JC	9.192	0.815	0.774	0.111	0.128

Note: NPF = negative performance feedback, Pro-JC = promotion-oriented job crafting, $\chi^{2}$/*df* = chi-square/degrees of freedom, CFI = comparative fit index, TLI = Tucker–Lewis index, RMSEA = root mean square error of approximation, SRMR = standardized root mean square residual. Source: made by authors according to the questionnaires.

**Table 3:** Results of CFA for AI negative performance feedback model.

Models	Factors	$\chi^{2}$/*df*	CFI	TLI	RMSEA	SRMR
Four-factors model 2	AI NPF, Anger, Algorithm aversion, Pre-JC	2.851	0.942	0.933	0.059	0.046
Three-factors model 3	AI NPF, Anger + Algorithm aversion, Pre-JC	12.687	0.787	0.754	0.133	0.128
Three-factors model 4	AI NPF, Anger, Algorithm aversion + Pre-JC	13.452	0.774	0.738	0.137	0.151
Two-factors model 3	AI NPF + Pre-JC, Anger + Algorithm aversion	21.644	0.619	0.566	0.177	0.170
Two-factors model 4	AI NPF + Anger, Algorithm aversion + Pre-JC	22.902	0.596	0.540	0.182	0.205
One-factor model 2	AI NPF + Anger + Algorithm aversion + Pre-JC	21.518	0.619	0.569	0.176	0.170

Note: NPF = negative performance feedback, Pre-JC = prevention-oriented job crafting; $\chi^{2}$/*df* = chi-square/degrees of freedom, CFI = comparative fit index, TLI = Tucker–Lewis index, RMSEA = root mean square error of approximation, SRMR = standardized root mean square residual. Source: made by authors according to the questionnaires.

**Table 4:** Regression results for the mechanism between leader negative performance feedback and promotion-oriented job crafting.

Variables	Shame			Promotion-Oriented Job Crafting		
	Model 1	Model 2	Model 3	Model 4	Model 5	Model 6
Gender	−0.130 ***	−0.082 *	−0.025	−0.168 ***	−0.110 ***	−0.053 **
Age	−0.061	0.095	0.124 *	−0.126 *	0.063	−0.004
Education	−0.133 ***	−0.041	−0.018	−0.139 ***	−0.028	0.001
Marriage	0.007	0.028	0.048	0.007	0.033	0.013
Working years	0.029	−0.021	−0.045	0.059	−0.001	0.014
Leader NPF		0.557 ***	0.570 ***		0.672 ***	0.282 ***
Leader trust			0.311 ***			
Shame						0.699 ***
Leader NPF × Leader trust			−0.402 ***			
F	5.420 ***	51.278 ***	57.265 ***	8.323 ***	95.722 ***	373.197 ***
R^2^	0.040	0.321	0.413	0.06	0.468	0.801

Note: * *p* < 0.05, ** *p* < 0.01, *** *p* < 0.001; NPF = negative performance feedback, F = F-statistic, R^2^ = coefficient of determination. Source: made by authors according to the questionnaires.

**Table 5:** Regression results for the mechanism between AI negative performance feedback and prevention-oriented job crafting.

Variables	Anger			Prevention-Oriented Job Crafting		
	Model 7	Model 8	Model 9	Model 10	Model 11	Model 12
Gender	−0.030	−0.042	−0.060	−0.006	−0.018	0.018
Age	0.261 ***	0.208 ***	0.188 ***	0.256 ***	0.203 ***	0.024
Education	0.176 ***	0.102 **	0.080 *	0.158 ***	0.084 *	−0.004
Marriage	−0.028	−0.042	−0.059	0.010	−0.004	0.032
Working years	−0.076	−0.048	−0.040	−0.083	−0.055	−0.014
AI NPF		0.282 ***	0.160 ***		0.281 ***	0.038
Algorithm aversion			−0.060			
Anger						0.862 ***
AI NPF × Algorithm aversion			0.239 ***			
F	9.282 ***	17.398 ***	18.819 ***	9.104 ***	17.197 ***	324.919 ***
R^2^	0.066	0.138	0.188	0.065	0.137	0.777

Note: * *p* < 0.05, ** *p* < 0.01, *** *p* < 0.001; NPF = Negative performance feedback, F = F-statistic, R^2^ = coefficient of determination. Source: made by authors according to the questionnaires.

**Table 6:** Bootstrap (=5000 times) results at different levels of leader trust.

Leader Trust	Mediating Effect of Shame	Boot SE	Boot LLCI	Boot ULCI
−0.845 (low)	0.453	0.029	0.397	0.510
0.000 (medium)	0.311	0.023	0.264	0.355
0.845 (high)	0.170	0.028	0.112	0.223

Source: made by authors according to the questionnaires.

**Table 7:** Bootstrap (=5000 times) result at different levels of algorithm aversion.

Algorithm Aversion	Mediating Effect of Anger	Boot SE	Boot LLCI	Boot ULCI
−0.508 (low)	−0.033	0.052	−0.137	0.067
0.000 (medium)	0.129	0.043	0.044	0.209
0.508 (high)	0.290	0.046	0.199	0.378

Source: made by authors according to the questionnaires.

## Data Availability

The data presented in this study are available on request from the corresponding author due to privacy and ethical restrictions.

## Appendix A

The reading materials for both the leader and the AI group were identical across conditions, with the only variation being the source of negative performance feedback. Li Ming for the leader group and the “AX-300” AI evaluation system for the AI group.

**Reading Materials**
Leader Negative Performance Feedback. Li Ming, head of the performance appraisal team at a leading technology company, was responsible for conducting monthly evaluations of all software engineers. The entire process was independently managed by Li Ming and his team, who were solely accountable for the outcomes. The appraisal considered multiple aspects of employee performance, including project delivery, task completion efficiency, and overall work quality. AI Negative Performance Feedback. Participants in this condition read that the company had recently implemented the “AX-300” AI evaluation system, a data-driven system designed to conduct monthly reviews of employee performance. The system automatically integrated a range of indicators, such as project delivery, task completion efficiency, and accuracy of outputs, to provide a rigorous and systematic evaluation. The entire process was executed independently by the AI, which held exclusive authority over the results.
During a recent monthly assessment, 200 engineers participated in the monthly review. Based on a series of evaluations, and according to the decisions made by Li Ming’s team/the “AX-300” AI evaluation system, ten employees were classified as “below standard,” consistent with the company’s policy that roughly 5% of staff should be flagged each month for insufficient performance. The evaluation results were announced promptly on the company’s internal platform, yet no additional details, such as individual scores, evaluation criteria, or explanatory notes, were disclosed. According to the assessment outcomes reported by Li Ming’s team/the “AX-300” AI evaluation system, one employee, Chen Yu, was identified as failing to meet expectations. Compared with colleagues who had successfully managed multiple key projects and produced notable technical contributions, Chen Yu’s performance over the past month reflected deficiencies in both technical execution and professional confidence.
